# Supplementary material for: A pan-cancer analysis of homeobox family: expression characteristics and latent significance in prognosis and immune microenvironment
Source: Front Oncol. 2025 Feb 6;15:1521652. doi: 10.3389/fonc.2025.1521652 (PMC11840236; doi:10.3389/fonc.2025.1521652)
Supplement: Supplementary Figure 1 — Mutations of each HOX gene in 32 cancers (TCGA, PanCancer Atlas) by cBioportal. [file DataSheet1.zip › Suppl.files/raw code.zip!/code/4.survival/survivalplot.zip!/survivalplot/HOXA3_OS.SKCM.pdf]

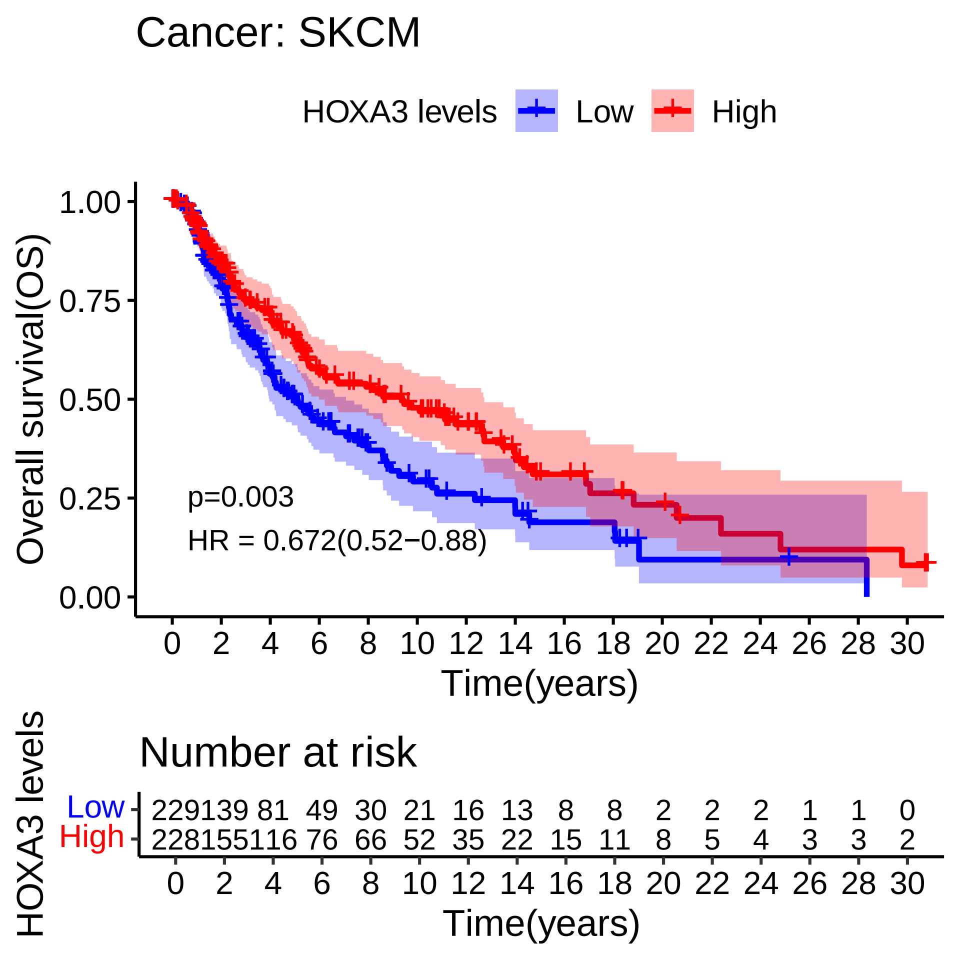

Cancer: SKCM
HOXA3 levels
Low
High
1.00
0.75
0.50
0.25
0.00
Overall survival(OS)
p=0.003
HR = 0.672(0.52−0.88)
0 2 4 6 8 10 12 14 16 18 20 22 24 26 28 30
Time(years)
Number at risk
HOXA3 levels
Low 229 139 81 49 30 21 16 13 8 8 2 2 2 1 1 0
High 228 155 116 76 66 52 35 22 15 11 8 5 4 3 3 2
0 2 4 6 8 10 12 14 16 18 20 22 24 26 28 30
Time(years)
